# Hex Me If You Can

**DOI:** 10.1111/cgf.14608

**Published:** 2022-10-06

**Authors:** P.‐A. Beaufort, M. Reberol, D. Kalmykov, H. Liu, F. Ledoux, D. Bommes

**Affiliations:** ^1^ University of Bern; ^2^ Université catholique de Louvain; ^3^ CEA; ^4^ Université Paris‐Saclay

**Keywords:** CCS Concepts, • ***General and reference*** → ***Evaluation***, • ***Computing methodologies*** → ***Mesh geometry models***, • ***Information systems*** → ***Test collections***

## Abstract

HexMe consists of 189 tetrahedral meshes with tagged features and a workflow to generate them. The primary purpose of HexMe meshes is to enable consistent and practically meaningful evaluation of hexahedral meshing algorithms and related techniques, specifically regarding the correct meshing of specified feature points, curves, and surfaces. The tetrahedral meshes have been generated with Gmsh, starting from 63 computer‐aided design (CAD) models from various databases. To highlight and label the diverse and challenging aspects of hexahedral mesh generation, the CAD models are classified into three categories: simple, nasty, and industrial. For each CAD model, we provide three kinds of tetrahedral meshes (uniform, curvature‐adapted, and box‐embedded). The mesh generation pipeline is defined with the help of Snakemake, a modern workflow management system, which allows us to specify a fully automated, extensible, and sustainable workflow. It is possible to download the whole dataset or select individual meshes by browsing the online catalog. The HexMe dataset is built with evolution in mind and prepared for future developments. A public GitHub repository hosts the HexMe workflow, where external contributions and future releases are possible and encouraged. We demonstrate the value of HexMe by exploring the robustness limitations of state‐of‐the‐art frame‐field‐based hexahedral meshing algorithm. Only for 19 of 189 tagged tetrahedral inputs all feature entities are meshed correctly, while the average success rates are 70.9% / 48.5% / 34.6% for feature points/curves/surfaces.

## 1. Introduction

**Figure 2:**
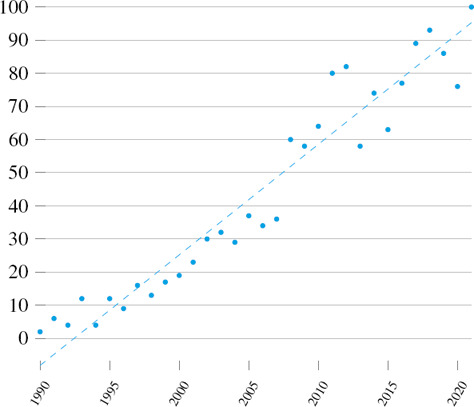
Yearly number of publications related to hexahedral meshing. Source: app.dimension.ai (criteria: hexahedral mesh, in title and abstract)

In the last decades, the meshing community has been actively working on developing algorithms and tools, in order to implement a robust and automatic hexahedral mesher, c.f. Figure 2. Although numerous attemps have been conducted, there is still no satisfactory solution up to this day. In fact, there is still no *automatic* method *robustly* generating *high‐quality* hexahedral meshes for general shapes. Industrial methods manage to provide high‐quality meshes, but they typically involve tedious and lengthy user interventions. Automatic methods such as advancing front [BRM∗14] and polycube‐based [CAS∗19] approaches are neither guaranteed to be robust, nor assured to build high‐quality hexahedra. Solely, octree‐based procedures [GSP19] can automatically and robustly supply a full hexahedral mesh, but with a quality far from ideal close to features (points/curves/surfaces). Three‐dimensional octahedral frame‐based methods are a promising candidate to automatically generate high‐quality meshes. However, they are lacking robustness due to non‐meshable frame‐field topology [LZC∗18], and other robustness issues in the generation of volumetric integer‐grid maps [PCS∗22].

**Figure:**
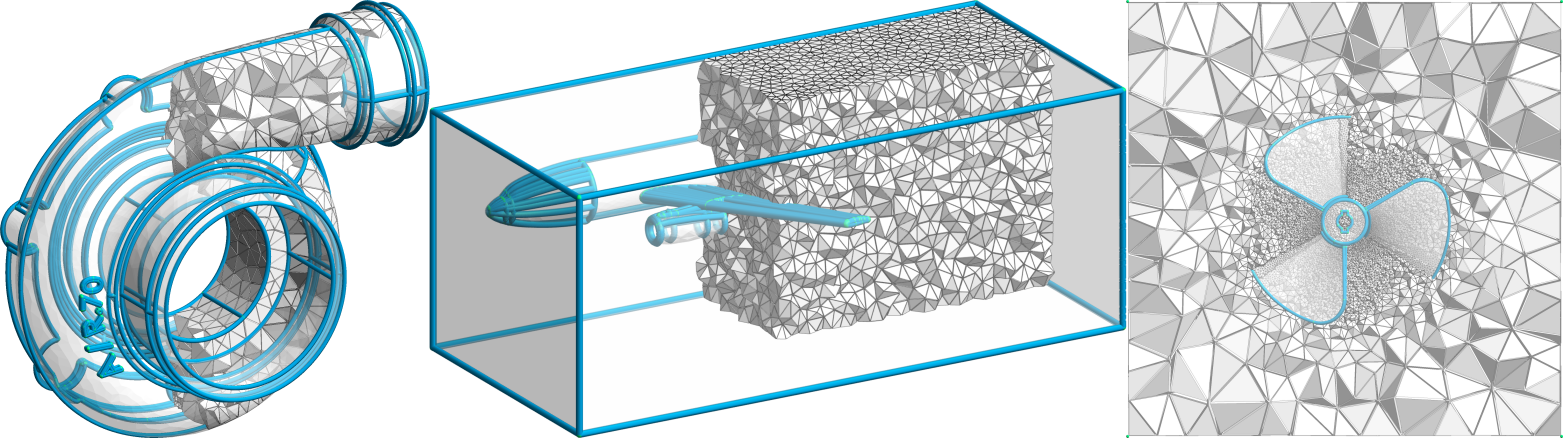
Three example models of HexMe (https://hexme.algohex.eu): The tetrahedral meshes faithfully represent feature points, curves (depicted in blue), and surfaces of the underlying CAD primitives.

Recently, [BTP∗19] has introduced HexaLab to the meshing community. HexaLab is a visualization tool enabling the evaluation of hexahedral meshes by filtering elements and showing various quality metrics (corner scaled Jacobian, edge ratio, etc.). In addition, HexaLab collects the *ouput* hexahedral meshes of various state‐of‐the‐art methods. However, there is no common dataset of *input* models, complicating the comparison of different methods.

Most of the algorithms tackling the hex meshing challenge use tetrahedral meshes as input or during some intermediate steps. However, there is no suitable tetrahedral dataset for objective analysis and meaningful comparison of hexahedral mesh generators. Our goal is to provide such a dataset to reveal common robustness issues and guide future research toward practical relevance.

**Figure 3:**
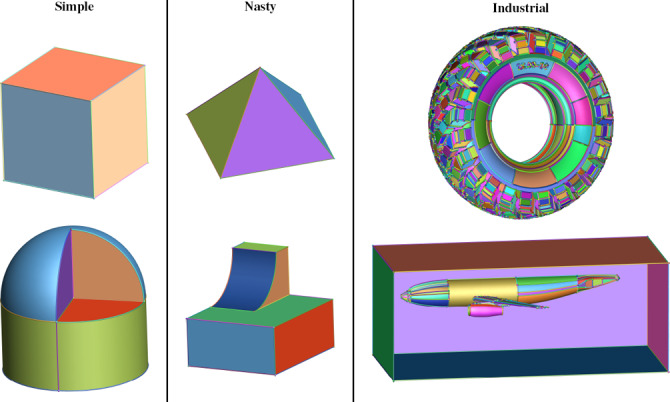
HexMe uses three categories of CAD models: simple, nasty and industrial.

The result of our endeavour is the HexMe dataset, a collection of tetrahedral meshes with tagged feature entities. The feature entities are special points, curves and/or surfaces, which need to be accurately captured by a hexahedral mesh, c.f. Fig.3. Please note that such feature points/curves/surfaces are common in all mesh generation scenarios, where they impose corresponding constraints on the local structure of the desired mesh. All meshes have been generated from computer‐aided design (CAD) models, following a workflow defined with Snakemake [MJL∗21], using the Gmsh [GR09] API with custom parameters defined in yaml metadata files. CAD models are classified into three categories (simple, nasty, industrial), in order to grade their difficulty and consistency. For each model, three meshes are provided: two resolutions (curvature‐adapted, uniform) to analyze the mesh dependency of algorithms, and an embedding of the object into a box resulting in interior feature structures. The meshes including the feature tags are exported as .vtk datafiles (version 2, ASCII mode), a mesh format which is broadly used and easily accessible.

HexMe and HexaLab share the goal of guiding future research on hexahedral meshing algorithms – HexMe through a suitable dataset of *input* models, HexaLab through the analysis and comparison of *output* hexahedral meshes.

The HexMe dataset has been designed to meet the following goals:

(*𝒢*0) **Unambiguous & Ready‐to‐use** – The dataset offers volumetric tetrahedral meshes, ready to be used by any method relying on a tet mesh. Different methods can be compared without any bias introduced by ambiguous conversion procedures as for instance from surface or CAD representations.
(*𝒢* ^1^) **Challenging** – The dataset specifically includes *nasty* geometric configurations that are often avoided, as well as real‐world *industrial* models containing an assembly of such difficulties.
(*𝒢*2) **Discriminative** – The dataset allows to diagnose and grade the limitations of hexahedral algorithms. There are *simple* models enabling a sanity check, whereas each *nasty* model is designed to reveal robustness w.r.t. a specific type of difficulty. The tessellation dependence of a method is evaluated by including two different tetrahedral meshes for each model.
(*𝒢*3) **Realistic** – The dataset mimics the workflow of a numerical practitioner, where all feature entities have to be preserved through all stages of the mesh generation pipeline. Explicitly defining such constraints is essential for comparison of methods since often it is possible to significantly improve the mesh quality or simplify the meshing task by violating some feature constraints. Consequently, output statistics are only comparable and meaningful if identical constraints have been enforced in the mesh generation.
(*𝒢*4) **General** – So far, most hex meshing methods have been evaluated only on individual objects with a single boundary. This is only a special case of the more challenging general volumetric meshing problem with arbitrary interior structures that arise for instance when simulating multi‐material scenarios like fluid‐structure interaction. Consequently, we include general test cases with interior feature constraints by embedding CAD models in a bounding *box.*
(*𝒢*5) **Sustainable** – We intentionally limit the number of models to restrict the evaluation to challenging and meaningful geometries. This is important since hex meshing algorithm often include costly mixed‐integer optimization and evaluating with thousands or millions of complex inputs is computationally infeasible. Moreover, the workflow has been designed in a sustainable manner, such that changes of the workflow require solely the regeneration of the affected models (Snake‐make cache).
(*𝒢*6) **Mutable** – Simultaneously to the algorithms, the set of challenging and meaningful geometries will change over time. HexMe is designed in a way that new models and even meshing definitions can be added conveniently (Snakemake workflow available on a public GitHub). We envision that the entire mesh generation community will actively contribute to future versions of HexMe.

In the following, we first describe in Section 2 datasets related to HexMe and discuss their differences. Afterwards, Section 3 introduces the pipeline to produce the tetrahedral meshes from CAD models, and Section 4 summarizes the content of HexMe. Finally, in Section 5, a state‐of‐the‐art frame‐field‐based hexahedral meshing pipeline is applied to the dataset to verify that HexMe is suitable to benchmark robustness of hex meshing algorithms.

## 2. Related Datatsets

There is a shift among the scientific community. Open science that is readily reproducible and shared is becoming popular. Among other things, this is mainly possible thanks to the free access to datasets supporting this open research. Consequently, an increased number of datasets has been published recently, where in the area of computer graphics ShapeNet [CFG∗15], ModelNet [WSK∗15], and Fusion360 [WPL∗21] are popular resources. In the following, we present the five datasets in more detail that are most related to HexMe and discuss their similarities and differences.

**Tetwild** Even though Tetwild [HZG∗18] is a tetrahedral meshing technique, it is also a tetrahedral dataset, since the authors provide the output of their algorithm applied to Thingi10k [ZJ16], a triangular dataset. This tetrahedral dataset is the tetrahedrization of ten thousand models from Thingi10k. The tetrahedral meshes are msh2 binary files, with a scalar per tetrahedron exposing the minimal dihedral angle. The 10k meshes are stored on Google Drive, within an archive tar.gz (∼9.5GB).

**ABC** The ABC [KMJ∗19] dataset is a collection of one million computer‐aided design models for geometric deep learning. All CAD files are from Onshape, and the original information related to those models is recorded within a metadata file *meta.*yml. Some processing tasks are done in order to filter the duplicate and broken models. The filtered models *para.*zip are afterwards converted into .step and .stl files using Parasolid. Gmsh [GR09] is then used to provide either uniform or curvature‐adapted triangular meshes, which are exported as .obj meshes from the .step files. Differential quantities are stored in those .obj files, while the vertices and triangles of the mesh are respectively matched to the feature curves and patches, through another metadata file *feat.*yml. Further files may be provided, depending on the success of the processing. The dataset is downloadable by chunks containing 7z archives of above files.

**Thingi10k** Historically, Thingi10k [ZJ16] is the first dataset providing ten thousand diverse, complex and quality .stl triangulations of 3D (printing) models. All models come from Thingiverse, and have been selected only if they were tagged *featured* by the Thingiverse staff. An online query interface is provided, which returns all the contextual and original information related to a .stl triangulation. It is also possible to download the whole dataset as an archive tar.gz from Google Drive (∼9GB).

**SimJEB** The recent SimJEB [WBM21] dataset provides 381 tetrahedral meshes from CAD models, by following a *semi‐automated* pipeline. The CAD models come from a challenge organized by GrabCAD. Those former 700 models have been filtered (mostly based on the filename), manually repaired, and then meshed using the commercial software HyperMesh. A structural simulation was performed using the commercial software OptiStruct. The 381 .vtk tetrahedral meshes surviving this pipeline are hosted through the Harvard Dataverse (∼1.6GB), along with the corresponding clean CAD .stp file, triangular surface .obj meshes, finite element .fem models, and simulation .csv results. The final models are identified by an integer, specified by *readme* files. A web page allows to browse the designs, and to explore the data.

[GSP19] Recent grid‐based hexahedral meshing papers [GLYL20, PLC∗21, LPC21] have benchmarked their method on a common dataset gathered by [GSP19]. This dataset consists of 202 .obj triangular meshes from [FBL16] and Drexel CAD repository (2018), 109 of them are equipped with feature curves. The feature curves have been extracted using a dihedral angle of 140 degrees in combination with manual adjustment. A .fgraph file specifies the set of feature edges but without grouping them semantically into feature curves. The 202 triangular meshes with the 109 feature annotations are available as a supplementary .zip file (∼1.4GB).

The available datasets differ by (i) their selection of models, (ii) whether triangular or tetrahedral meshes are supplied, (iii) potential specification of feature entities (0D/1D/2D/3D), and (iv) their infrastucture to access models and to potentially contribute new models. Table 1 summarizes the comparison between HexMe and the related datasets. Please note that for datasets solely providing triangular surface meshes, we interpret these as a 2D feature specification, even if no explicit tags are available.

**Table 1:** Summary of datasets related to HexMe.

Dataset	Database	Mesher	Mesh Tri / Tet	Format	Features 0D|1D|2D|3D	Interface
Thingi10k	Thingiverse	‐	Tri	.stl	×|×|✓|×	query engine
TetWild	Thingi10k	TetWild	Tet	.msh2	×|×|×|×	Google drive
ABC	Onshape	Gmsh	Tri	.obj	×|✓|✓|×	chunks
SimJEB	GrabCAD	HyperMesh^©^	Te t	.vtk	×|×|×|×	webpage
[GSP19]	[FBL16] Drexel cad repository	‐	Tri	.obj	✓|✓|✓|×	.zip
**HexMe**	ABC GrabCAD MAMBO (crafted)	Gmsh	Tet	.vtk	‖|✓|✓|✓|✓	web catalog

SimJEB is *by‐design* the closest dataset to HexMe: small number of tetrahedral meshes from CAD models, semi‐automated meshing pipeline, web catalog and .vtk mesh format. However, all SimJEB geometries describe a jet engine bracket, while HexMe supplies more diverse and challenging types of geometries. ABC is the only related dataset providing meshes with feature tags corresponding to the CAD geometry. However, the ABC dataset consists only of triangular surface meshes and does not define any volumetric discretization. The dataset of [GSP19] supplies 109 triangular surface meshes with annotated feature points and curves. But neither tetrahedral meshes, nor tags relating feature entities to the corresponding CAD primitives, or a sustainable and extensible workflow are available. The Thingi10k and Tetwild meshes have not been generated from CAD files and thus do not specify feature entities.

## 3. From CAD to Tets

All the tetrahedral meshes provided by HexMe have been produced from three categories of CAD models, Figure 3:

- simple models: basic shapes that are assumed to be easily hexmeshable, i.e. the target hexahedral topology is fair, e.g., a cube (s01o_cube.geo), or a cut hemisphere on a cylinder (s10o_cyl_cutsphere.stp).
- nasty models: academic shapes that are challenging to hex‐mesh, e.g., a pyramid [VPR19] (n09o_pyramid.geo), or a ski jump (n02o_skijump_anti_box_cyl.geo).
- industrial models: lifelike shapes, which hexahedrization is highly valuable for numerical simulation, e.g., a truck tire (i28o_gc_tire_1218.step from GrabCAD), or an aircraft for CFD (i31o_dlr_f6.brep [VTM∗08,BERF08]).

The 63 CAD models have been selected to cover known configurations that are challenging for current hexahedral meshing algorithms. In the future, the dataset will evolve together with the algorithms with the goal of providing a minimal set of test cases, which is maximally meaningful.

In contrast to the comparable datasets (Section 2), the CAD models come from several databases: ABC dataset (originally from Onshape), GrabCAD and MAMBO. Some of the CAD models were created specifically for HexMe, using Gmsh and Siemens NX software. Those latter models are released to the *public domain*, while the other ones are regulated by licenses, which are respectively: Onshape Terms 1(g)(i), GrabCAD Terms (c.f. related FAQ) and Apache 2.0. The above information is reported within a metadata file (with the following nomenclature (s|n|i) (\d{2})o_{extra}.yaml) per CAD model with a short description of the shape, e.g. i28o_gc_tire_1218.yaml:

**Table:** 

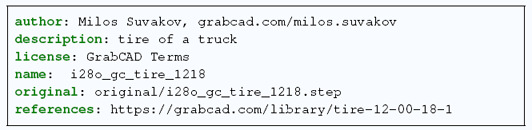

**Figure 4:**
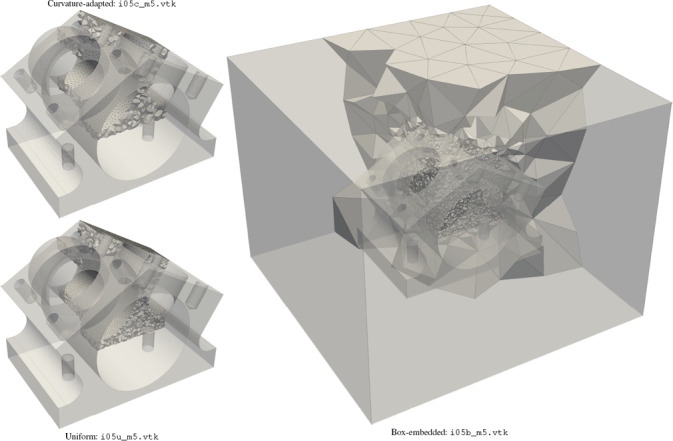
There are three tetrahedral meshes per CAD model, e.g. i05o_m5.step from MAMBO.

For each CAD model, three tetrahedral meshes are provided, c.f. Figure 4:

- **c**urvature‐adapted: the mesh element size is adapted to the curvature, and upper bounded such that the CAD geometry is sufficiently preserved (e.g. i05c_m5.vtk).
- **u**niform: the mesh element size is constant, even in the neighborhood of the tiniest geometrical features (e.g. i05u_m5.vtk).
- **b**ox‐embedded: the initial model is embedded in a box that is twice as large as the original bounding box, and the corresponding mesh is generated such that the smallest gap between the box limits and the initial model is meshed by one layer of tetrahedra (e.g. i05b_m5.vtk).

The pipeline handling the mesh generation is orchestrated by Snakemake [MJL∗21], a popular (currently ∼ 7 citations per week) scalable workflow management system. In a few words, Snakemake is a modern version of GNU Make, whose syntax is close to Python. The workflow Snakemesh consists of two rules. The first rule meshes defines which meshes should be produced. The second rule cad2vtk generates a mesh from a CAD model and a metadata file (s|n|i)(\d{2})(c|u|b)_{extra}.yaml containing the custom mesh options (for **c**urvature‐adapted, **u**niform, or **b**oxembedded). To do so, this second rule runs a python script using Gmsh API, with a maximum of 8 threads. For each mesh, a log file (s|n|i)(\d{2})(c|b|u)_{extra}.txt is written with the corresponding console output, in order to record the history of the meshing task.

Snakemake scans the workflow in a backward fashion, meaning that the input files are determined from the output ones. In other words, the purpose of the first rule is to state all meshes that should be produced. Afterwards, the second rule provides those meshes by identifying the corresponding input files accordingly, which are the CAD model and the metadata file. This backward identification is the key of the workflow definition, since the rules are mostly written with wildcards. The use of Snakemake easily yields a sustainable dataset, since a rule is applied only if an output is either missing or older than the corresponding input.

Gmsh [GR09] does not only mesh the volume, but also the feature entities as defined by the CAD model. In addition to the tetrahedral elements, there are triangle, edge and vertex elements (lower dimensional elements are conforming to the higher ones) to respectively discretize feature surfaces, feature curves and feature points. Those features are identified by the CAD with a *tag* (i.e. a positive integer), which corresponds to a physical group within Gmsh. Doing so, the corresponding mesh elements are created accordingly with the inherited CAD tag. Meshes are exported as vtk Datafile Version 2.0, in ASCII mode. The used Gmsh git‐version is written within the file header. A mesh is defined as an UNSTRUC‐TURED_GRID, with the four following sections:

1. POINTS: coordinates of every node
2. CELLS: number of nodes and nodal definition of every element (vertices, edges, triangles and tetrahedra); the second number on the cell section is the total number of integer values
3. CELL_TYPES: integer corresponding to the element type {1:vertex, 3:edge, 5:triangle, 10:tetrahedron}
4. CELL_DATA: integer corresponding to the element tag that belongs to the CAD feature.

**Table:** 

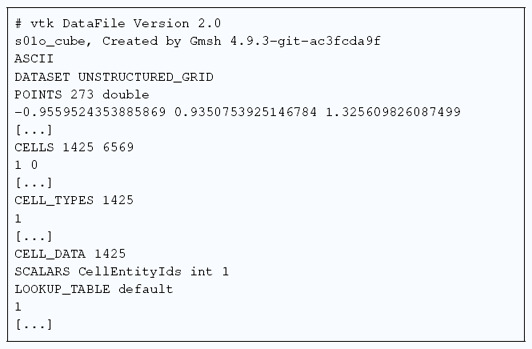

Overall, there are 189 meshes, whose filenames follow the nomenclature (s|n|i)(\d{2})(c|u|b)_{extra}.vtk, which summarizes the corresponding model (s|n|i)(\d{2}) and mesh (c|u|b) types.

## 4. HexMe Anatomy

The HexMe tetrahedral dataset is downloadable in a single file: hexme.zip (∼1.5GB). Alternatively, it is possible to download meshes *one‐by‐one* from the catalog (the catalog is mostly generated by Snakemake, using the report feature) The catalog is split into three categories (i, n, s), that correspond to the model categories (respectively: industrial, **n**asty, simple). Within each category, there are three subcategories (b, c, u), that correspond to the mesh types (respectively: **b**ox‐embedded, **c**urvature‐adapted, **u**niform).

**Figure 5:**
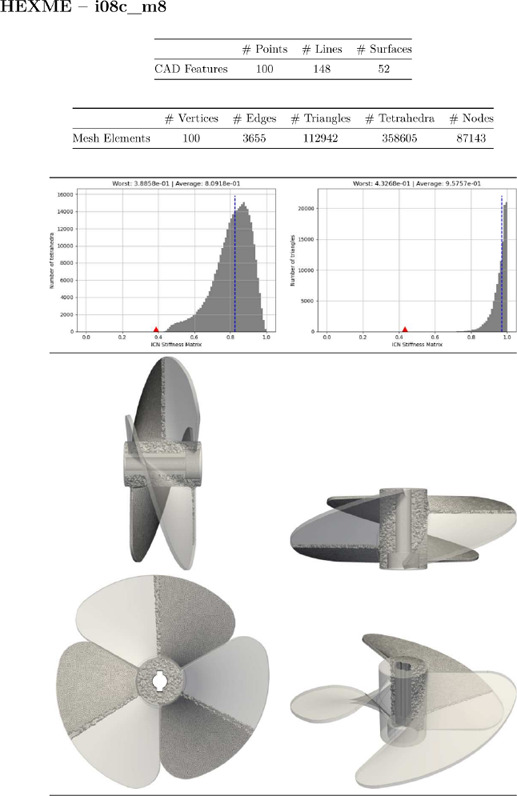
Sheet summarizing i08c_m8.

An entry of the catalog is described by two pictures (a cut view and a quality histogram), a .pdf file, a .vtk mesh, the corresponding log file (s|n|i)(\d{2})(c|u|b)_{extra}.txt and the metadata file (s|n|i)(\d{2})o_{extra}.yaml related to the CAD model. A summary of the mesh is also available in a .pdf sheet, Figure 5. The summary provides topological information about the CAD model (number of points, curves and surfaces) and the mesh (number of vertices, edges, triangles, tetrahedra and nodes). Moreover, two histograms related to the inverse condition number (ICN) [JGTR16, §2.1] of triangles and tetrahedra are plotted. Finally, four screenshots (*xy*‐, *yz*‐, *zx*‐ and 3D‐views) of the cut mesh are displayed.

On top of HexMe catalog, there is a GitHub page hosting all the necessary input files to run the workflow. The tetrahedral meshes are not hosted on this git repository (the git history would be too heavy otherwise). The main purpose of this git repository is to expose the workflow that has been used for the mesh generation. Through this repository, it is possible to report an issue if a mesh does not meet user expectations. The meshing community is invited to actively contribute to the HexMe dataset, by creating pull‐requests for proposing new models and/or filtering of existing ones. There will be releases with appropriate git‐tag, whenever the dataset has been significantly updated.

**How to contribute.** The workflow supports CAD models with extensions .geo, .step, .stp, or .brep. For each CAD model, four metadata files need to be defined. The first one specifies general information about the model

**Table:** 

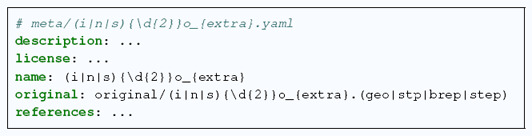

The other three files define the desired meshing parameters for each mesh type (c|u|b), respectively:

**Table:** 

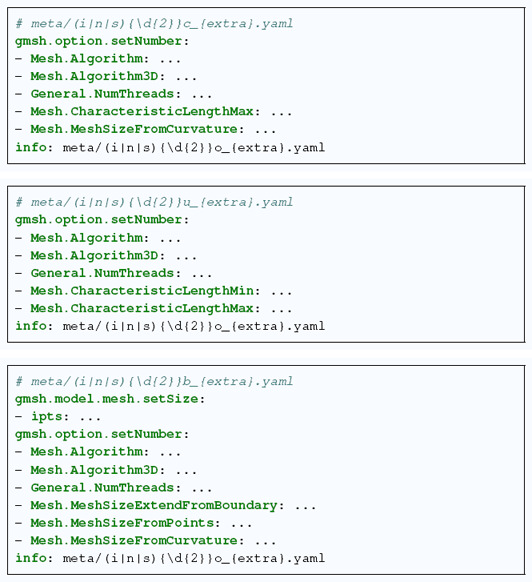

The items of the section gmsh.option.setNumber correspond to the options that Gmsh provides. The items Mesh.CharacteristicLength∗ constrain the mesh element size range. In the case of a uniform mesh, those values are chosen identically. For further details about Gmsh options, we refer the reader to the corresponding documentation. The info entry links the mesh metadata to the metadata of the CAD model. Finally, observe that the box‐embedded metadata has a section related to gmsh.model.mesh.setSize. The value of ipts is the mesh element size used for the interior of the CAD model (we recommend to use the element size of the uniform meshing case by default). For such box‐embedded meshes, the outer part of the model is meshed with one layer of tetrahedra in the smallest gap between the model boundary and the limits of the box.

## 5. Example Evaluation

To demonstrate the value of HexMe, we challenge a *state‐of‐the‐art* pipeline of frame‐field based hexahedral meshing with our dataset and evaluate its robustness. Robustness issues regularly stem from hard constraints induced by feature points, curves and surfaces. Consequently, our primary concern here is to quantify how faithful the tagged input features are reproduced in the generated hexahedral meshes. Note that this is a novel way to assess robustness, which is more fine‐grained than simply counting *passed or failed* per model. It is also more meaningful in our setting than other straightforward choices (percentage of hexahedral elements, or distortion of the integer‐grid map for instance) where excellent numbers could be reported despite some feature constraints are violated.

For the same reason, typical quality metrics (such as the scaled Jacobian) have been omitted since they are meaningless for state‐of‐the‐art algorithms, where most of the generated hexahedral meshes are incomplete. Obtaining a high quality hexahedral mesh of a subregion is often significantly easier if features are not preserved. However, there is no doubt that quality metrics will be important in the future of HexMe as soon as the robustness of available algorithms reaches a sufficient level.

The frame‐field based hex‐meshing pipeline consists of the following steps:

1. Determination of the target edge length *h*
2. Specification of frame‐field alignment constraints.
3. Feature‐aligned smooth frame‐field generation. [RSL16]
4. Integer‐Grid map generation guided by the frame‐field. [NRP11]
5. Hexahedral mesh extraction from the IG map. [LBK16]
6. Verification of feature points, curves, and surfaces.

While upon success the above algorithm delivers promising hexmesh quality, it can neither guarantee a valid integer‐grid map, nor a valid hexahedral mesh. Failures are caused by (i) non‐meshable frame‐field topologies, (ii) non‐robust integer‐quantization, or (iii) the inability to guarantee local injectivity for volumetric maps, see [PCS∗22] for more details. Since all of the above defects are frequently triggered by feature constraints, HexMe is well‐suited to quantify the lack of robustness of the candidate pipeline. Please note that we did not include singularity repair strategies based on collapses [LLX∗12] or splits [JHW∗14], since their repair capabilites are comparable to what HexEx [LBK16] is able to handle throughout the extraction step. The above hex‐meshing pipeline is released as an open‐source C++ library to enable reproduction and ease comparison for future research.

Before presenting and discussing the results of our evaluation, we describe in more detail the choice of the target edge length (Step 1), frame‐field alignment constraints (Step 2), and the verification of feature entities (Step 6). The verification of represented features requires special attention since the integer‐grid map might contain degeneracies that are repaired by HexEx but prevent a trivial transfer of feature tags from the tetrahedral to the hexahedral mesh.

**Target Edge Length.** We determine the target edge length *h* of the hexahedral mesh such that *n* hexahedra are generated. Considering that the volume of the unit cube is one, we obtain 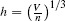, with *V* being the volume of the input tetrahedral mesh. For all our experiments we choose *n* = 50*k.*

**Frame‐Field Alignment Constraints.** Frame‐fields can be seen as a continuous relaxation of an integer‐grid map, cf. [PCS∗22]. A frame exhibits the same symmetries as a cube and therefore represents the local rotation of a cube. Consequently, we require that the frames align tangentially to all feature curves and feature surfaces. In a HexMe tet mesh, a feature curve is represented by a 1‐manifold chain of edges. For each of the interior vertices along such a chain, we estimate a tangent vector by averaging the two incident edges and then use it as an alignment constraint for the frame‐field. Similarly, a feature surface is represented by a 2‐manifold subset of triangles and we compute (area‐weighted) vertex normals that are also used as alignment constraints.

**Figure 6:**
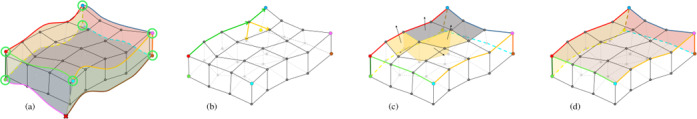
Feature matching process: feature points (a), feature curves (b), feature patches (c), and the final result (d).

**Verification of Feature Points.** For each feature point of the input tet mesh, we search the closest vertex in the hex mesh. If the distance is below τ = *h*, the feature point is counted as correctly reproduced, otherwise as invalid, as illustrated in Fig. 6 (a). Please note that we do not require higher‐accuracy geometric re‐produciton of features but merely want to assess whether the connectivity of the hex mesh is able to represent the feature.

**Verification of Feature Curves.** In HexMe, a feature curve always contains a feature point. Consequently, we can verify feature curves by a greedy path search from a source feature vertex to a target feature vertex in the hexahedral mesh. At the source vertex we begin with the edge most parallel to the feature curve tangent at the corresponding point in the tetrahedral mesh. Then we extend the path through subsequent vertices by always following the next edge, which is most parallel to the previous one, as illustrated in Fig. 6 (b). This simple geometric heuristic is motivated by the fact that feature curves represent smooth 1‐manifolds, and turned out to be reliable in our experiments. A feature curve is successfully verified if the path reaches the target vertex, while all intermediate points have a distance below τ. In all other cases, we classify the feature curve as not being reproduced correctly. Please note that whenever the source or target feature point are not verified in the previous stage, the feature curve verification will automatically fail.

**Verification of Feature Surfaces.** For verification of feature surfaces we use a geometrically guided strategy similar to the one for feature curves. Starting from a seed quadrilateral, we grow the surface in a breadth‐first manner to neighboring elements based on normal similarity, as illustrated in Fig. 6 (c). We ensure 2‐manifoldness by disallowing growth that would result in more than 2 incident quadrilaterals at one edges. The growth procedure terminates when newly added elements reach a distance above τ to the input feature surface, or when it encounters edges belonging to feature curves. The resulting surface is correctly reproduced if its boundary feature curves coincide with those specified in the input tetrahedral mesh. Again, a feature surface can only be correctly reproduced, if all its incident feature curves are already correctly reproduced.

**Results and Discussion.** We run the hexahedral mesh generation pipeline on all HexMe models and count the percentages of correctly reproduced feature points, curves, and surfaces. A table of full statistics can be found in the supplement. Only for 19 out of 189 models all feature entities are reproduced correctly, while the average success rates are 70.9% / 48.5% / 34.6% for feature points/curves/surfaces.

**Figure 7:**
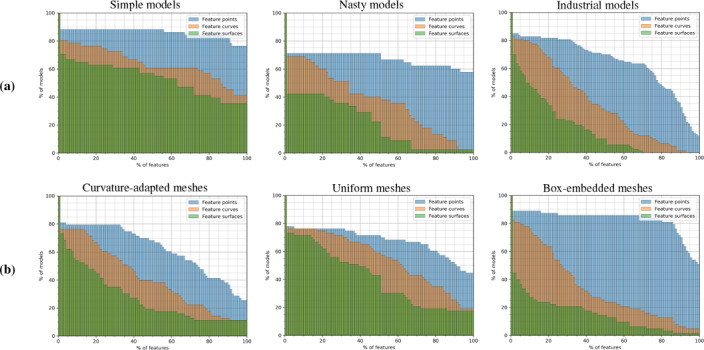
Cumulative histograms of the percentage of (a) models and (b) meshes, in respect to the percentage of tetrahedral features that are recovered in the hexahedral meshes. Observe that in the case of the box‐embedded mesh type, the features of the embedding box (8 points, 12 curves and 6 surfaces) are discarded.

Figure 7 (a) shows the cumulative percentage of simple/nasty/industrial models where *at least* a certain percentage of feature points/curves/surfaces in the tetrahedral mesh (of any type, boxembedded/curvature‐adapted/uniform) are present in the corresponding hexahedral mesh. As expected, the hexahedral pipeline is performing the best on the category of simple models. Around 50% of the hexahedral meshes preserve at least 100% / 86% / 63% of the feature points/curves/surfaces of the input tetrahedral meshes. For the meshes of the nasty models, those figures drop to 100% / 34% / 1%, while for the meshes of the industrial models they are only 78% / 32% / 8%.

Figure 7 (b) provides similar information, but for the curvature‐adapted, uniform and box‐embedded meshes of all models. In the case of the box‐embedded meshes, the feature verification does not count the features corresponding to the box (8 points, 12 curves, 6 surfaces), since those features are trivially recovered. The hexahedral pipeline performs better for uniform meshes than for curvature‐adapted. While integer‐grid map based approaches do not necessarily require a dense tetrahedral mesh in their domain, they explicitly require mesh vertices and edges to represent singularities. Locally inadequately coarse meshes can therefore lead to collapses in the singularity structure that induce global nonmeshability and consequently worsen feature reproduction. The inner features of the box‐embedded meshes are clearly more challenging to preserve compared to the uniform and curvature‐adapted meshes.

**Figure 8:**
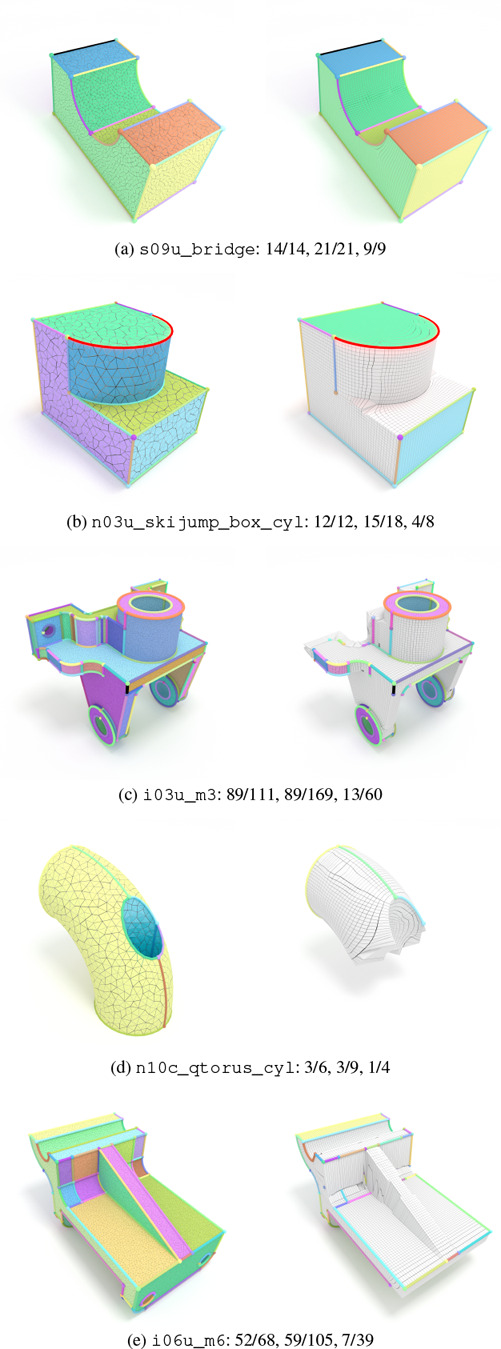
Example input tet meshes (left) and output hex meshes (right). The numbers indicate ratios of correctly reproduced feature points, curves and surfaces, as additionally depicted visually.

Figure 8 visualizes five representative examples of HexMe tet meshes and their corresponding generated hex meshes. Feature points, curves and surfaces are color‐coded in the tet meshes. The corresponding feature entities in the hexahedral mesh are only color‐coded if reproduced correctly. Here we solely provide general statistics and some qualitative examples. A complete enumeration and categorization of defects are out of the scope of this evaluation but will be an important task for future work.

## 6. Conclusion

The contributions of HexMe are twofold. On the one hand, it is a tetrahedral dataset with tagged feature entities and on the other hand, it is a transparent workflow. The main objective of HexMe is to provide tetrahedral meshes for the meaningful assessment of hexahedral meshers and associated auxiliary tools such as 3D frame‐fields. In the future, the choice of meshes will evolve together with the progress of hexahedral meshing techniques. Therefore, we publish the full workflow to ensure that the HexMe dataset can be updated easily and does not become outdated in the future.

The selected 63 CAD models come from several databases. Their origin and license are recorded within a metadata file. There are three categories of CAD models, and three types of meshes per CAD model. The 189 meshes are produced thanks to a workflow that is defined with Snakemake. The CAD features are reproduced by Gmsh as lower dimensional elements (vertices, edges, triangles), with corresponding tags. The meshes are expressed as .vtk Datafile Version 2.0 in ASCII mode.

There are two ways to access the HexMe tetrahedral meshes – either by downloading all of them in a 1.5GB .zip file, or by picking individual ones from the catalog. In addition to the meshes and log files, the catalog yields the metadata related to the CAD model, a summary about the mesh, and information related to the workflow. The files that are involved in the workflow, are available on a GitHub repository. From this git repository, it is possible to raise issues and/or pull requests to improve the dataset or the workflow.

The commit corresponding to the version used for this paper has been tagged HexMe‐1.0. The dataset has been uploaded to Zenodo, and can be referenced with the following doi 10.5281/zenodo.6642020. Whenever a new release occurs, those version tags will be updated accordingly. Those taggings are crucial in order to keep track of the assessment of hexahedral methods.

## Acknowledgements

This project has received funding from the European Research Council (ERC) under the European Union's Horizon 2020 research and innovation programme (AlgoHex, grant agreement No 853343). Open access funding provided by Universitat Bern.

## Supporting information

Supplement Material
